# In vivo synergy of radiation and hu14.18-IL2 immunocytokine results in a memory T cell response in a syngeneic murine melanoma model

**DOI:** 10.1186/2051-1426-2-S3-P160

**Published:** 2014-11-06

**Authors:** Zachary S Morris, Emily Guy, David Francis, Monica Gressett, Richard Yang, Alexander Rakhmilevich, Jaquelyn Hank, Stephen Gillies, Paul Harari, Paul Sondel

**Affiliations:** 1 University of Wisconsin, Madison, WI, USA; 2 Provenance Biopharmaceuticals, Carlisle, USA

## Purpose

Tumor-specific monoclonal antibodies (mAb) are a common type of immunotherapy capable of engaging innate immune cells to elicit antibody-dependent cell-mediated cytotoxicity (ADCC). We recently demonstrated in vivo synergy between radiation (RT) and ADCC using the anti-GD2 hu14.18 mAb. We now investigate the potential of hu14.18-IL2 immunocytokine (IC) to augment this synergy.

## Method and materials

C57BL/6 mice were engrafted with syngeneic GD2-expressing B78 melanoma. Macroscopic tumors (~ 200 mm3) were treated with sham or single fraction 12 Gy RT. Mice then received 5 daily intra-tumor injections of human IgG, hu14.18 mAb, or hu14.18-IL2 IC. NK or T cell depletion was achieved by intraperitoneal injection of a depleting mAb (NK1.1 or CD4/CD8 mAbs). After 90 days mice rendered disease-free by initial treatment were re-challenged with a second injection of B78 melanoma. After an additional 30 days mice not developing tumors were injected at distinct sites with syngeneic B16 melanoma (related to B78 but lacking GD2) and Panc02 pancreatic cancer cells.

## Results

**Figure 1:**
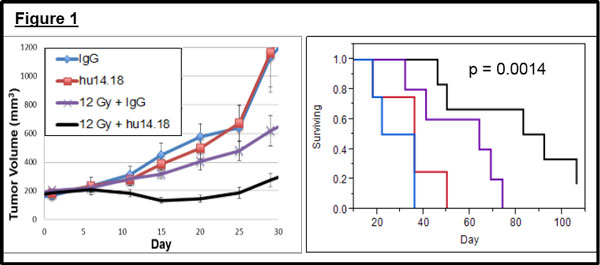
**In vivo synergy of radiation and hu14.18K322A in a murine melanoma tumor model**.

**Figure 2:**
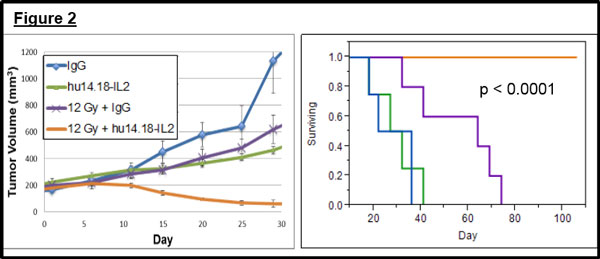
Enhanced in vivo synergy elicited by radiation and hu14.18-IL2 immunocytokine in a murine melanoma tumor model.

In tumor-bearing mice we observe synergy between RT and anti-GD2 mAb resulting in tumor regression and improved animal survival (Figure 1). This interaction of RT and hu14.18 is inhibited by depletion of NK cells and is not observed in mice that lack Fcϒ receptors. Synergy is markedly enhanced by substituting mAb with hu14.18-IL2 (Figure 2), resulting in durable complete resolution of tumors in 71% (22/31) of animals. This synergy of RT and IC is minimally affected by NK cell depletion but is largely abrogated in nude mice and Fcϒ receptor-deficient mice. Immunohistochemistry on post-treatment tumor specimens demonstrates enhanced recruitment of CD8+ T cells to tumors following combined treatment with RT and IC. Of animals rendered disease-free following combined treatment, 90% (18/20) did not grow a tumor following a second injection with B78 cells while no age-matched control mice (0/23) rejected these cells. A subset of mice rendered disease-free following initial treatment was later depleted of T cells and none (0/5) rejected repeat engraftment with B78 cells. Among mice rendered disease-free by initial treatment and remaining disease-free after B78 re-challenge, 75% (9/12) rejected subsequent engraftment with related GD2-deficient B16 melanoma cells. None of these rejected simultaneous injection of Panc02 cells and no age-matched controls (0/11) rejected the same B16 cells.

## Conclusion

We present evidence of synergy between RT and hu14.18-IL2 resulting in a memory T cell response. Our findings suggest a therapeutic opportunity for combining RT with immunotherapies that simultaneously target innate immune response and T-cell activation.

